# Early vocational rehabilitation and psychological support for trauma patients to improve return to work (the ROWTATE trial): study protocol for an individually randomised controlled multicentre pragmatic trial

**DOI:** 10.1186/s13063-024-08183-w

**Published:** 2024-07-02

**Authors:** Denise Kendrick, Rebecca Lindley, Lauren Blackburn, Cristina Roadevin, Ellen Thompson, Isabel Andrews, Fahim Anwar, Adam Brooks, Edd Carlton, Robert Crouch, Florence Day, Steve Fallon, Amanda Farrin, Laura Graham, Karen Hoffman, Rebekah Howell, Jain Holmes, Marilyn James, Trevor Jones, Blerina Kellezi, Jade Kettlewell, Richard Morriss, Roshan das Nair, Davina Richardson, Matthew Smith, Stephen Timmons, Alexandra Wright-Hughes, Kathryn Radford

**Affiliations:** 1 Centre for Academic Primary Care, Lifespan and Population Health, School of Medicine, University Park, Nottingham, NG7 2RD UK; 2 Nottingham Clinical Trials Unit, University Park, Nottingham, NG7 2RD UK; 3 Centre for Rehabilitation & Ageing Research (CRAR), Injury, Recovery Sciences and Inflammation (IRIS), School of Medicine, Medical School, University of Nottingham, Nottingham, NG7 2UH UK; 4 Centre for Health Innovation, Leadership and Learning, Nottingham University Business School, Nottingham, NG8 1BB UK; 5 Institute of Mental Health, Innovation Park, Triumph Road, Nottingham, NG7 2TU UK; 6 Health Division, SINTEF, Trondheim, Norway; 7 Imperial College Healthcare NHS Trust, The Bays, South Wharf Road, London, W2 1NY UK; 8 Academic Department of Rehabilitation Medicine, Leeds General Infirmary, Leeds, LS1 3EX UK; 9 Cambridge University Hospital NHS Foundation Trust, Addenbrooke’s Hospital, Cambridge, UK; 10 North Bristol NHS Trust Southmead Hospital, Southmead Road, Westbury-On-Trym, Bristol, BS10 5NB UK; 11 University Hospital Southampton NHS Foundation Trust, Tremona Road, Southampton, S016 6YD UK; 12 Royal Victoria Infirmary, Queen Victoria Road, Newcastle Upon Tyne, NE1 4LP UK; 13 East Midlands Major Trauma Centre, Nottingham University Hospitals NHS Trust, Nottingham, NG7 2UH UK; 14 Department of Psychology, Nottingham Trent University, 50 Shakespeare Street, Nottingham, NG1 4FQ UK; 15 Centre for Trauma Sciences, Barts Health NHS Trust and Queen Mary University London, Blizard Institute, 4 Newark St, London, E1 2AT UK; 16 Leeds Institute of Clinical Trials Research, University of Leeds, Leeds, LS2 9JT UK

**Keywords:** Trauma, Injury, Vocational rehabilitation, Psychology, Return to work, Employment, Quality of life, Health economics, Occupational therapy

## Abstract

**Background:**

Moderately severe or major trauma (injury severity score (ISS) > 8) is common, often resulting in physical and psychological problems and leading to difficulties in returning to work. Vocational rehabilitation (VR) can improve return to work/education in some injuries (e.g. traumatic brain and spinal cord injury), but evidence is lacking for other moderately severe or major trauma.

**Methods:**

ROWTATE is an individually randomised controlled multicentre pragmatic trial of early VR and psychological support in trauma patients. It includes an internal pilot, economic evaluation, a process evaluation and an implementation study. Participants will be screened for eligibility and recruited within 12 weeks of admission to eight major trauma centres in England. A total of 722 participants with ISS > 8 will be randomised 1:1 to VR and psychological support (where needed, following psychological screening) plus usual care or to usual care alone. The ROWTATE VR intervention will be provided within 2 weeks of study recruitment by occupational therapists and where needed, by clinical psychologists. It will be individually tailored and provided for ≤ 12 months, dependent on participant need. Baseline assessment will collect data on demographics, injury details, work/education status, cognitive impairment, anxiety, depression, post-traumatic distress, disability, recovery expectations, financial stress and health-related quality of life. Participants will be followed up by postal/telephone/online questionnaires at 3, 6 and 12 months post-randomisation. The primary objective is to establish whether the ROWTATE VR intervention plus usual care is more effective than usual care alone for improving participants’ self-reported return to work/education for at least 80% of pre-injury hours at 12 months post-randomisation. Secondary outcomes include other work outcomes (e.g. hours of work/education, time to return to work/education, sickness absence), depression, anxiety, post-traumatic distress, work self-efficacy, financial stress, purpose in life, health-related quality of life and healthcare/personal resource use. The process evaluation and implementation study will be described elsewhere.

**Discussion:**

This trial will provide robust evidence regarding a VR intervention for a major trauma population. Evidence of a clinically and cost-effective VR intervention will be important for commissioners and providers to enable adoption of VR services for this large and important group of patients within the NHS.

**Trial registration:**

ISRCTN: 43115471. Registered 27/07/2021.

**Supplementary Information:**

The online version contains supplementary material available at 10.1186/s13063-024-08183-w.

## Introduction

### Background and rationale {6a}

In England in 2020/21, 268,000 working age adults were admitted to hospital following injury [1]. Whilst many patients recover from their injuries, [2] disabilities are common amongst those who survive [3]. In addition to the physical consequences of injury, patients often report a negative impact on mental health [4, 5] and long-term reductions in quality of life [6, 7]. High NHS resource use and associated cost for this patient group has also been reported [8].

Trauma care in England underwent an extensive re-organisation in 2012 with the establishment of Major Trauma Centres (MTCs) to provide care for patients with moderately severe or major trauma (injury severity score (ISS) ≥ 9), who have a higher risk of mortality and/or of permanent disability [9]. The establishment of MTCs has been associated with a significant improvement in survival [10]. Improved survival has led to an increased number of patients requiring rehabilitation to enable return to activities that are meaningful to them and to society, including return to work. To address these needs, patients with an ISS > 8 are required to have a written rehabilitation prescription [9]. However, a recent systematic review [6] found that one third of patients who experienced major trauma had not returned to work after 1 year. Patients with more severe injuries are less likely to return to work than patients with less severe injuries and have greater productivity losses [11]. The economic impact of injuries on patients and their families can be extensive, including loss of individual earnings, reduction in household income, increase in social security benefits and increased time away from the workplace for caregivers [3, 11–14]. Not returning to work after injury is also associated with poorer health status and quality of life [15, 16]. In those that do return to work, employment stability can be threatened by mental health problems such as depression, anxiety and post-traumatic distress [17, 18]. Whilst the specific benefits of being employed vary from person to person, being in work is associated with better overall health and wellbeing for the individual [19]. Being in work also reduces economic burden and the risk of poverty and allows individuals to feel a valued part of society [20].

Returning to education post-injury also presents challenges. A systematic review of students’ perspectives on returning to education after traumatic brain injury (TBI) identified cognitive, emotional, physical and psychosocial difficulties in returning to education. The impacts of these difficulties included needing to change courses, educational or vocational goals or reduce workload as well as school avoidance or leaving school at an earlier age than planned. A lack of understanding by schools of TBI, limited awareness of student needs and failure to provide necessary accommodations were also reported [21].

Vocational rehabilitation (VR) is “a multi-professional approach that is provided to individuals of working age with health-related impairments, limitations, or restrictions with work functioning and whose primary aim is to optimise work participation” [22]. The majority of research on the effectiveness of VR focuses on specific injury cohorts which limits generalisability to patients with other moderate or severe trauma. There is some evidence that VR interventions can improve rates of RTW in patients with specific traumatic injuries (e.g. spinal cord and brain injuries), [23–27] particularly those that are individually tailored, are case coordinated, set vocational goals and engage with employers [24–26, 28–32]. Recent UK research shows that VR is not widely available or accessible for many with moderately severe or major trauma, particularly those with musculoskeletal injuries [33]. This paper describes the protocol for a randomised controlled trial evaluating the clinical and cost-effectiveness of specialist VR intervention plus usual care delivered by an occupational therapist (OT) and with input from a clinical psychologist (CP) where needed (based on psychological screening), amongst those with moderately severe or major trauma (ISS > 8).

### Objectives {7}

The primary objective of this study is to establish whether the ROWTATE VR intervention plus usual care is more effective than usual NHS rehabilitation alone for improving participants’ self-reported work outcome (return to work or education of at least 80% of pre-injury hours) at 12 months post-randomisation. The secondary objectives will be measured at 3, 6 and 12 months post-randomisation and will determine:

- Whether the intervention leads to improvement in other self-reported work/education outcomes (including number of hours returned to work, percentage of pre-injury hours returned to work, intentions, retention, job changes, time to return, retirement and sickness absence).
- Whether the intervention improves psychological wellbeing, work self-efficacy, financial impact of injury, purpose in life and health-related quality of life.
- Whether the intervention changes overall health and social care resource use.
- The cost-effectiveness of the intervention compared to usual care alone.

### Trial design {8}

This trial protocol has been reported in line with the Standard Protocol Items: Recommendations for Interventional Trials (SPIRIT) checklist [34].

ROWTATE is a prospective, open-label, pragmatic, multi-centre, superiority, individually (1:1) randomised controlled trial (RCT) with two parallel groups. It has an embedded internal pilot, and also includes economic and process evaluations and an implementation study. The internal pilot will include all eight MTCs and will assess whether progression criteria thresholds are met for recruitment rates after 10 months of recruitment and for retention rates 12 months later (see the “Recruitment” section for further details).

Participants in the intervention arm will receive an individually tailored specialist VR intervention delivered by trained OTs and CPs (where needed) in addition to usual care. Participants in the control arm will receive usual care only. Usual care may be provided by primary care, secondary care or local authority services, such as Social Services and other community-based services provided by private companies and charitable organisations.

## Methods: participants, interventions, outcomes

### Study setting {9}

Participants will be recruited from eight MTCs which are geographically dispersed across the UK.

### Participant eligibility criteria {10}

#### Inclusion criteria

Those who meet all of the following criteria at time of screening will be eligible for inclusion:

- Aged 16–69 years at time of injury
- Admitted to an MTC within the last 12 weeks
- ISS > 8 at admission (i.e. moderately severe or major trauma)
- In work (including self-employed and voluntary work) or in full-time education at time of injury. Full-time education is defined as “education undertaken in pursuit of a course, where an average of more than 12 h per week is spent during term time receiving tuition, engaging in practical work, receiving supervised study, or taking examinations” [35].
- No plans to retire within the next year
- Have sufficient proficiency in English to contribute to the data collection required for research or be willing to use an approved interpreting service for data collection
- Resident in MTC catchment area
- Able to provide informed consent

#### Exclusion criteria

Those meeting the following criteria will not be eligible for inclusion:

- No fixed address
- Returned to work/voluntary work/education for ≥ 80% of pre-injury hours

Participation in other research studies may preclude participation in the ROWTATE trial. Potential participants already enrolled in other research studies will be discussed with the Chief Investigators, and recruitment to the ROWTATE trial will be assessed on a case-by-case basis, taking into account similarities in interventions and outcomes and participant burden.

### Consent or assent {26)

#### Who will obtain informed consent or assent from potential trial participants or authorised surrogates, and how {26a)

Informed consent will be obtained prior to the participant undergoing study procedures. Consent will be taken in person or remotely via video or phone call. The principal investigator (PI) or their nominee will explain study details and provide the information sheet (see supplementary online material for consent forms and participant information sheets), either in person or remotely. The PI or nominee will answer questions, ensuring the participant has sufficient time to consider their participation. If recruitment is conducted in person, written informed consent will be obtained. Where a participant is unable to sign or make a mark on the consent form, verbal consent will be obtained that will be witnessed by an independent observer (staff member, relative or friend). If recruitment is conducted remotely, verbal consent will be obtained and will be documented on the consent form.

Prior to consenting participants, their right to withdraw will be explained. They will be able to withdraw from different aspects of the study such as intervention, completion of questionnaires, receipt of text messages and/or access to medical records. They can withdraw for any reason at any time without their care being affected. Identifiable data already collected with consent will be retained and used in the final study analysis.

Patients lacking capacity to provide consent for themselves will not be included. In the event that a participant loses capacity to consent during the study, the participant will be withdrawn from the study. No further data will be collected, or any other research procedures carried out on, or in relation to the participant.

### Additional consent provisions for collection and use of participant data and biological specimens {26b}

Additional informed consent will be sought from the intervention arm participants and their employers, carers and general practitioners and OTs, CPs, mentors and commissioners to take part in interviews and/or observations as part of the embedded process evaluation and implementation study. Further details will be reported in the ROWTATE process evaluation and implementation study protocol.

## Interventions

### Explanation for choice of comparators {6b}

The comparator for the intervention is usual care. This is defined as “the wide range of care that is provided in a community whether it is adequate or not, without a normative judgement” [36]. Usual care may be provided by primary care, secondary care, community and social services and determined by local policies and practices. Participants may also access care provided by the private sector as part of usual care. Usual care will be available to both intervention and usual care only participants.

To increase external validity and relevance of trial findings to clinical practice, we will use an unrestricted usual care approach, whereby the trial protocol does not restrict access to usual care, in line with our pragmatic trial design and the possibility for heterogeneity of usual care treatments available for people who have suffered injury [37].

### Intervention description {11a}

Participants will be randomised to receive either the ROWTATE VR intervention plus usual care, or usual care alone.

#### ROWTATE VR intervention (plus usual care)

The ROWTATE VR intervention is individually tailored specialist VR that aims to lessen the impact of injury on work/education demands by assessing the participant’s role as an employee/student to find acceptable strategies to overcome problems. It will be delivered by OTs and, where needed, by CPs, who will be trained by an experienced OT and CP to deliver the intervention. The intervention will be delivered predominantly by remote methods (video call, phone), with face-to-face contact where required (e.g. participant requests or therapist considers face to face contact is necessary, limitations to technology use).

The training package comprises:

- The intervention manual
- Training materials emailed to OTs and CPs that include relevant publications, a video presentation about the study design, normal and abnormal responses to trauma and a case study to complete.
- Two half-day online workshops that cover study design, research contamination, tele-rehabilitation and the roles of the OTs and CPs.
- Two day intervention workshop, using blended online and face-to-face delivery. The workshop covers trial design, minimising contamination, intervention delivery and data collection using role-play, clinical scenarios and case studies to support learning.
- One day refresher workshop approximately six months after site recruitment commences
- Monthly group mentoring provided by an experienced OT and CP, with ad hoc individual support (e.g. email/phone) provided between sessions, for the trial duration.

Competency to deliver the intervention will be assessed by a team objective structured clinical examination (TOSCE) with an individual and a team written task, following the 2-day workshop. The TOSCE will be video-recorded and observed and assessed by experienced OTs and CPs who were not involved with training the ROWTATE therapists. The written task will be measured against expert-agreed model answers. Further details of the competency assessment will be provided in the protocol for the embedded process evaluation and will be published elsewhere.

The intervention will ideally commence within 2 weeks of randomisation. Content, dose, intensity and duration (up to 12 months post-randomisation) will be individually tailored based on participant’s needs, preferences and specific employment contexts. The intervention will include:

- Assessing the impact of the injury on the participant, family and the participant’s role as a worker
- Setting and reviewing vocational goals
- Educating participants, employers and families about the effects of the injury and its impact on work and find acceptable strategies to lessen that impact
- Monitoring and adjusting the participant’s post-injury life and work goals
- Preparing participants for work by establishing structured routines with gradually increased activity levels and opportunity to practice work skills
- Liaising with relevant stakeholders such as employers, employment advisors (e.g. occupational health), solicitors and the healthcare team to advise about the effects of the injury and to plan and monitor a phased return to work
- Routine monitoring of mood and emotional issues, via routine use of questionnaires, observation and responses during clinical sessions, by the OT and, if needed, the CP

The need for CP involvement will be determined by the OT screening for mental health problems using validated measures (Patient Health Questionaire-9 (PHQ-9) [38], Generalised Anxiety Disorder Assessment-7 (GAD-7) [39], 15-item Impact of Events Scale (IES-15) [40], Whooley depression screening questions [41], Patient Health Questionnaire-Panic Disorder (PHQ-PD) [42]) at the initial intervention assessment and again at 6 months post-randomisation. Following a stepped care approach [43], participants scoring in the “moderate” range on psychological measures will be monitored by the OT for 1 month and if no improvement will be discussed with or referred to the CP. Participants scoring in the “severe” range on psychological measures will be referred to the CP for psychological assessment and support as needed. OTs can also discuss or refer participants to the CP who do not score in the moderate or severe range on psychological measures if they consider this clinically necessary.

The CP will deliver individualised psychological assessment and/or treatment, including evidence-based approaches, based on National Insatiate for Health and Care Excellence (NICE) recommendations for management of trauma-related mental health issues such as anxiety, depression and post-traumatic stress disorder (PTSD) [44–47], or facilitate the receipt of psychological assessment and/or treatment by referring the participant to appropriate usual care providers. The ROWTATE CP will have an important role alongside the OT in identifying barriers to return to work, as well as best therapeutic interventions to deal with these. Where ongoing cognitive or emotional issues need to be taken into account at the time of initial return to work, the CP should be involved in discussions regarding timing of and structure of the return to work process. In such cases, the CP may need to be involved in the monitoring of the return to work and respond to any new issues that arise requiring alteration to the return to work plan, or require any additional psychotherapeutic, or other mental health interventions, by the ROWTATE CP or others. Additionally, the CP will provide consultancy around psychological intervention to the OT and to other stakeholders.

### Criteria for discontinuing or modifying allocated interventions Modifications {11b}

Participants can discontinue and/or withdraw from the study intervention at any time (see {26a}) and will follow the same data collection follow-up schedule unless they withdraw from such aspects of the study.

### Strategies to improve adherence to interventions {11c}

The training, competency assessment and monthly mentoring provided to OTs and CPs aim to improve adherence to the intervention manual.

Participant adherence will be assessed by the number of sessions offered and attended and drop out prior to the agreed ending of intervention. This will be collected via a content CRF completed by the trial OT/CP at each intervention session. Non-compliance will be determined by no attendance at the first intervention session; poor compliance will be defined as attendance at < 30% of the offered intervention sessions or drop out prior to agreed ending of intervention; good compliance will be defined as attendance at ≥ 70% of offered intervention sessions with an agreed ending of the intervention.

Adherence to the intervention manual will be monitored using mentoring records, intervention content case report forms (CRFs) and other research intervention records. Using data from these sources, a fidelity checklist will be completed for all therapist-participant contacts for one randomly selected participant from a purposive sample of 20 OTs and from all CPs (there will be a smaller number of CPs than OTs, hence fidelity assessment will take place for all CPs). All therapist-participant contacts will be included for each randomly selected participant so that the entire participant intervention journey is assessed. Reasons for non-adherence will be documented (where possible) in mentoring records and factors moderating adherence will be identified from the intervention content CRFs and therapists’ research intervention records and recorded on the fidelity checklist.

Observations of therapist-participant intervention sessions will be piloted for feasibility with five therapist-participant sessions and if feasible to continue, one therapist-participant session will be observed from a purposive sample of 20 OTs and from all CPs. OTs will be purposively sampled to ensure representation from all sites for the fidelity checklist completion and for the observations. Where possible, the same OTs and CPs will be used for the fidelity checklist completion and the observations.

Further details of monitoring adherence and fidelity assessment will be provided in the process evaluation protocol.

### Concomitant care {11d}

No restrictions will be imposed on usual care or any other concomitant care or interventions during the study period.

### Provisions for post-trial care {30}

The OT will assess for ongoing needs at the end of the ROWTATE VR intervention and direct patients to suitable sources of support that are available to meet those needs.

### Outcomes {12}

#### Primary outcome measure

The difference between the two randomised groups in the proportion of participants who have returned to work at 12 month post-randomisation. This is defined as returning to employment or self-employment, voluntary work or full-time education and working at ≥ 80% of pre-injury working hours. This outcome measure arose from a focus group of major trauma survivors comprising a diverse range of injury types and causes (e.g. road traffic, sports, workplace injuries and falls), including multiple trauma and also traumatic brain injury. It included both professional and “blue collar” occupations pre-injury, some had made a successful return to work and some had not. The group discussed what constituted successful return to work, including the number of hours that would constitute a successful return to work/education and our primary outcome was developed based on this. Return to at least 80% of pre-injury working hours has also been used in previous research with major trauma patients to define complete or nearly complete return to work [48, 49]. This will be measured using bespoke questions as described under data collection methods below.

#### Secondary outcome measures

Secondary outcome measures will be assessed at 3, 6 and 12 months post-randomisation (unless otherwise indicated) and include:

- Difference in the proportion of participants who have returned to at least 80% of pre-injury working hours at 3 and 6 months
- Difference in mean number and percentage of pre-injury hours returned to work, days of sickness absence
- Difference in the proportion of participants intending to return to work/education, intending to retire, with type of work/education changes
- Difference in time to return to work/education (measured using bespoke questions)
- Difference in mean psychological wellbeing scores (Patient Health Questionnaire-9 (PHQ-9) [38], Generalised Anxiety Disorder Assessment-7 (GAD-7) [39], 6-item Impact of Events Scale (IES-6) [50])
- Difference in mean work self-efficacy scores (2 items from the Workability Index [51]) at 12 months
- Difference in mean financial impact of injury score (3 items from the Financial Chronic Stress Scale [52])
- Difference in mean purpose in life score (4-item Purpose in Life Test – Short Form [53]) at 12 months

#### Health economics

- Difference in mean health-related quality of life (EQ-5D-5L [54]) at 3, 6 and 12 months
- Difference in resource implications and cost-effectiveness (bespoke questions on health and social care resource use) at 3, 6 and 12 months
- Cost-effectiveness and cost utility per participant at 12 months

### Participant timeline {13}

**Fig. 1:**
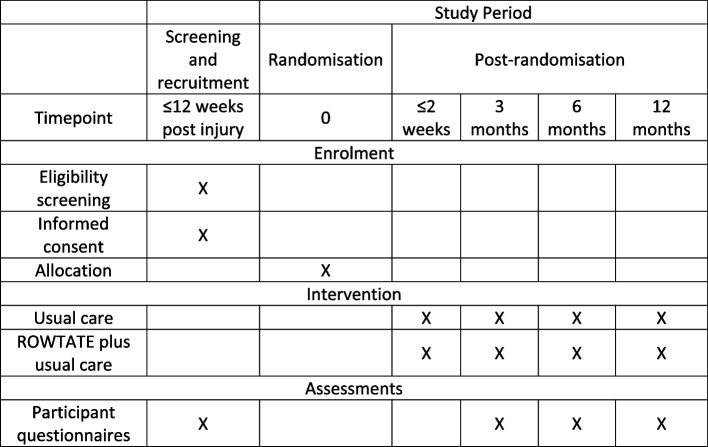
Timeline of screening, recruitment, randomisation, interventions and assessments

**Fig. 2:**
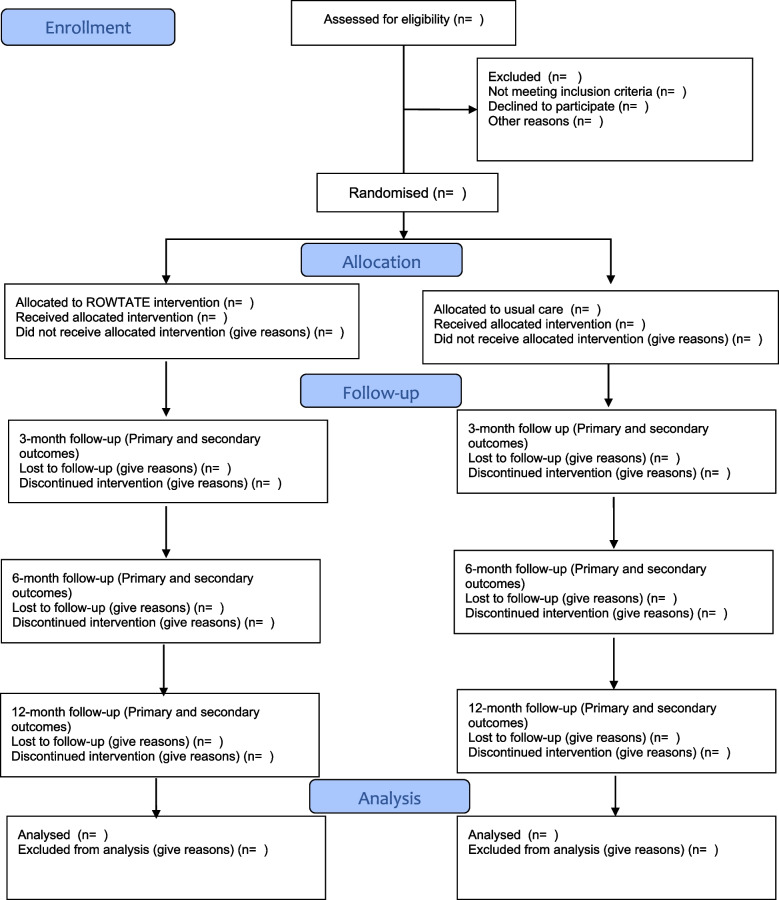
CONSORT flow diagram

Following admission to an MTC, patients will be screened for eligibility, their informed consent will be obtained and baseline assessments will be completed prior to randomisation. OTs will contact participants within two working days of randomisation to organise the initial assessment session, which should ideally take place within 2 weeks of randomisation. The intervention will continue up to 12 months post-randomisation, depending on participant need. The duration of usual care will vary between MTCs based on service provision at each site. Participants will complete follow-up questionnaires at 3, 6 and 12 months post-randomisation. A participant timeline (Fig. 1) and flow chart (Fig. 2) have been completed in accordance with SPIRIT Guidelines [34].

### Sample size {14}

The planned sample size for the study is 722 recruited participants equally distributed across usual care and ROWTATE VR intervention plus usual care. This will provide at least 90% power, at the 5% significance level, to detect a between-group 15% absolute difference in return to work rates, with an expected 60% return to work rate in the control arm, allowing for up to 30% loss to follow-up at 12 months and design effect up to 1.221. The design effect appropriately inflates the required sample size for a individually randomised controlled trial to account for clustering resulting from therapist-delivered interventions in both study arms. The design effect assumes an intra-cluster coefficient (ICC) less than or equal to 0.03 (80, 81); an expected cluster size (number of participants seen per therapist) of ~ 11 participants (~ 8 analysable participants) with four therapists per site in the intervention arm across eight sites, and a coefficient of variation in cluster size less than or equal to 0.25 (i.e. cluster sizes ranging 3–14 participants). The remaining therapists across the eight sites will provide usual care.

This sample size is sufficient with 93% power to detect a moderate 0.3 effect size between randomised groups in health-related quality of life measured by the EQ5D using the same assumptions as the primary outcome calculation. This is based on a standard deviation (SD) of 0.248 in those who had not returned to work and 0.176 in those who had returned to work obtained from unpublished data from our Impact of Injuries Study [55]. The minimal important difference for the EQ-5D has been estimated to be 0.074 [56].

#### Recruitment (15)

Potential participants will be identified at MTCs using a range of methods including bespoke trauma patient databases containing all patients with moderately severe or major trauma defined as an ISS > 8. Daily lists of inpatient trauma patients and daily trauma rounds, supplemented by knowledge of trauma/rehabilitation coordinators, ward occupational therapists and trauma case managers will assist in identifying appropriate patients.

Potential participants will be approached by their usual care team to introduce the study and assess eligibility. Those who are eligible will be given the participant information sheet (PIS) and an expression of interest form (EOI). If patients are discharged before they are approached, they will be phoned by the usual care team to discuss the study. They will be posted a study invitation, PIS and EOI form as well as receive an email if they have access to email. Those unable to be contacted via phone will receive a posted study invitation, PIS and EOI form. Those expressing interest will be contacted by the usual care team or a researcher to assess eligibility. An anonymised screening log will be used to monitor potentially eligible participants up to 12 weeks after admission to the MTC and returned monthly to the Clinical Trials Research Unit (CTRU) to aid identification of recruitment issues. Recruitment can take place either face-to-face or remotely via video call or phone. The target recruitment was at least six participants per MTC per month (after the first 3 months of recruitment) over a 20-month recruitment period, with staggered start dates for sites. Due to slower than anticipated recruitment, the recruitment target was subsequently amended to 32 participants per month across all eight MTCs and the recruitment period extended to 30 months, with an anticipated recruitment end date of 31/3/2024. Regular single-site and multisite recruitment meetings will be held to identify and respond to recruitment challenges.

All sites will participate in an internal pilot, which will assess the recruitment rate 10 months after the first two sites commence recruitment, and the retention rate 10 months later. The progression criteria are shown in Table 1. If any criteria are graded as amber, or another issue is identified which could affect successful trial completion, a rescue plan will be developed outlining steps to be taken to improve recruitment, and/or follow-up (as appropriate). This will be approved by the Trial Steering Committee (TSC) before submission to the National Institute for Health and Care Research (NIHR). If only red progression criteria are achieved, the Trial Management Group (TMG), TSC and funder will determine if the trial should be stopped.

**Table 1:** Progression criteria for the internal pilot

• Green (go): mean of more than 6 participants recruited per month per site; At least 80% of participants complete the primary outcome
• Amber (modify): mean of 4–6 participants recruited per month per site; 60–79% of participants complete the primary outcome
• Red (stop): mean of less than 4 participants per month per site; Less than 60% of participants complete the primary outcome

## Methods: assignment of interventions

### Allocation

#### Sequence generation {16a)

Individual participants will be randomly assigned to usual care or ROWTATE plus usual care in a 1:1 allocation ratio. Allocation will use a 24-h computer-generated minimisation programme incorporating a random element, with minimisation factors: Site, age (16-30, 31-45, 46-60, 61-69), sex (male, female), time since injury (0 to 4 weeks, 5–8 weeks, 9–12 weeks) and ISS (9–15, > 15).

#### Concealment mechanism {16b}

The online, central randomisation service will ensure allocation concealment, as allocation information will only be released after the patient has been consented and recruited into the trial, and after all baseline measurements have been completed.

#### Implementation {16c}

Randomisation will be requested by the staff member responsible for recruitment from the online, central randomisation service. An automated email will be sent from the randomisation service to the staff member responsible for recruitment, the site PI and the site’s intervention OTs confirming randomisation and the participant’s allocation. Research staff will inform the participant’s GP of study participation via letter. OTs will keep a record of all recruited participants to ensure they do not treat usual care participants. The Clinical Trials Research Unit (The CTRU) will inform participants via letter of their allocation with details of subsequent actions (e.g. follow-up assessments, OT contact).

### Blinding

#### Who will be blinded? {17a}

Participants and personnel delivering the intervention will not be blind to allocation group. To minimise the risk of detection bias, baseline data will be collected prior to participant randomisation. Researchers from other participating sites (not the recruitment site) who are blind to the allocation group will conduct priming calls prior to each follow-up time point to inform participants of upcoming questionnaires and to collect primary outcome data. If the researcher administering priming calls at the site becomes unblinded, they will be asked to inform CTRU as soon as possible and priming calls will be assigned to another researcher at the site.

#### Procedure for unblinding {17b}

Neither participants, the site researchers nor intervention delivery teams will be blinded to treatment allocation; therefore, there is no requirement for emergency unblinding procedures in this study.

## Methods: data collection and management

### Plans for assessment and collection of outcomes {18a}

Baseline assessments (Table 2) will be collected by clinical staff/researchers at site via face-to-face/telephone/video conferencing in hospital or following discharge as close as possible and prior to randomisation (within 12 weeks of admission to MTC).

**Table 2:** Baseline assessments

Assessment	Type	Method of completion
Participant demographics	Questionnaire booklet	Researcher/self-completion
Contact details	CRF	Researcher
Injury details	CRF	Researcher
Montreal Cognitive Assessment (MoCA)	CRF	Researcher
Work/education status	Questionnaire booklet	Researcher/self-completion
Health-related quality of life (EuroQol EQ-5D-5L)	Questionnaire booklet	Researcher/self-completion
Patient Health Questionnaire (PHQ-9)	Questionnaire booklet	Researcher/self-completion
Generalised Anxiety Disorder Assessment (GAD-7)	Questionnaire booklet	Researcher/self-completion
Impact of events scale (15 items)	Questionnaire booklet	Researcher/self-completion
World Health Organisation Disability Assessment Schedule 2.0 (12 items)	Questionnaire booklet	Researcher/self-completion
Recovery expectations	Questionnaire booklet	Researcher/self-completion
Financial Chronic Stress Scale (3 items)	Questionnaire booklet	Researcher/self-completion

*CRF* case report form

Follow-up assessments for primary, secondary and health economic outcomes will be measured by participant self-reported questionnaires at 3, 6 and 12 months post-randomisation (Table 3). This will be done either by telephone, postal or online completion using internet-based software (REDCap) depending on participant preference.

**Table 3:** Follow-up assessments

Assessment	Type	Method of completion	3 months	6 months	12 months
Discharge Information (including final ISS score)	CRF	Researcher	Quarterly		
Work/Education status	Questionnaire booklet	Researcher / Self-completion	X	X	X
Health-Related Quality of Life (EuroQol EQ-5D-5L)	Questionnaire booklet	Researcher / Self-completion	X	X	X
Patient Health Questionnaire (PHQ-9)	Questionnaire booklet	Researcher / Self-completion	X	X	X
Generalised Anxiety Disorder Assessment (GAD-7)	Questionnaire booklet	Researcher / Self-completion	X	X	X
Impact of Events Scale (6 item scale)	Questionnaire booklet	Researcher / Self-completion	X	X	X
Work Ability Index (WAI item 1 (workability scale) WAI item 2 (physical and mental demands)	Questionnaire booklet	Researcher / Self-completion			X
Financial Chronic Stress Scale (3 item scale)	Questionnaire booklet	Researcher / Self-completion	X	X	X
Purpose in life test—short form scale (4 items)	Questionnaire booklet	Researcher / Self-completion			X
Health and Social Care Resource Use (inpatient and outpatient hospital visits, primary and community care use, medication, aids and adaptations, informal care)	Questionnaire booklet	Researcher / Self-completion	X	X	X
Intervention Resource Use (intervention session content and additional activity forms recording therapist’s direct time and indirect time (time travelling to/from treatment appointments and administrative time)	CRF	Occupational therapists and clinical psychologists	Throughout intervention period		
Safety Reporting (workplace accidents requiring medical attention, workplace accidents involving equipment or adaptations to work environment, injuries received, healthcare used)	Questionnaire booklet	Researcher / Self-completion	X (only if RTW)	X (only if RTW)	X (only if RTW)

*RTW* return to work

#### Assessment instruments

The primary outcome of return to work for at least 80% of pre-injury hours will be captured using the following three questions:

1. Are you currently in work/ voluntary work/education in any capacity (including a phased return)?
2. Which of these statements currently applies to you?
  I am working
  I am doing voluntary work
  I am on a Government programme aimed at finding a job
  I am studying on a course
  I am on a Government programme aimed at finding a course
3. How many hours are you currently working or in education for each week?

Priming calls will be made to participants prior to administration of follow-up questionnaires and primary outcome data will also be collected, where possible at priming calls using the questions above. For those not contactable by priming calls, two-way SMS messaging will be sent asking participants to confirm work/education status (the primary outcome).

Secondary and health economic outcomes will be measured by a combination of bespoke questions and standardised tools. Bespoke questions will assess work/education intentions, time to return to work/education, retirement, sickness absence; and health and social care resource use. Outcomes measured using standardised tools include:

1. Health-Related Quality of Life using with the EuroQol EQ-5D-5L which measures self-reported health outcomes regarding mobility, self-care, usual activities, pain/discomfort and anxiety/depression [54]. It has been recommended by guidelines for use in comprehensive injury populations including injury of moderate severity and major trauma [57]
2. Psychological wellbeing using:
  PHQ-9 which is designed to screen for depression [38] in a variety of medical settings and is highly sensitive and specific [58]. It has been found to be a valid tool amongst acutely injured trauma survivors [59] and have high sensitivity, specificity and test–retest reliability in people with traumatic brain injury [60].
  GAD-7 which is used to screen for generalised anxiety disorders in a range of medical settings [39] and is highly sensitive and specific [61] and shows high internal consistency amongst people with TBI [62].
  IES-6 to measure post-traumatic distress symptoms. It has been shown to have high internal consistency, and high sensitivity and specificity with a cut-off of a mean score of 1.75 for a diagnoses of PTSD based on the Clinician Administered PTSD Scale [50]. It has also been shown to predict psychological distress in burn survivors at 6 months and 2 years post -injury [63].
3. Work self-efficacy using Work Ability Index which measures participants’ self-reported estimation of ability to do their work with respect to work demands, health and mental resources [51]. Items 1 (workability scale) and 2 (separate questions on physical and mental demands) will be used. Item 1 has been shown to predict sustainable RTW in construction workers with musculoskeletal conditions [64].
4. Financial impact of injury using Financial Chronic Stress Scale (3 item scale) which measures satisfaction with financial situation, difficulty meeting monthly bill payments and monthly finances [52].
5. Purpose in Life Test – Short Form which assesses a person’s perceived meaning and purpose in life. The scale contains 4 items measuring presence of goals, goal completion and meaning in life. Responses are on a Likert scale ranging from 1–7 with a total score ranging from 4 to 28. Higher scores on this scale are indicative of greater reported purpose in life. It has been shown to have acceptable reliability [53].

Research staff will receive training to ensure standardised completion of study-specific assessments.

### Plans to promote participant retention and complete follow-up {18b}

A range of retention strategies will be used, based on a systematic review of the evidence of methods to increase response rates [65] and designed in collaboration with our study PPI group. The retention strategies include:

- Collection of data enabling multiple methods of contacting participants (phone, email, post)
- Pre-notification of questionnaires using priming calls and SMS messaging
- Two reminders approximately 2 to 3 weeks later to non-responders, the first including the full questionnaire and the second a much shorter questionnaire collecting data only on the primary outcome and the EQ-5D-5L
- Thank you card and
- £10 gift voucher to thank participants for their time completing questionnaires

### Data management {19}

Data collection forms transferred to/from the CTRU will be coded with a trial number and will include two participant identifiers, the participant’s initials and date of birth. Study data will be held securely on paper and electronically at the University of Leeds CTRU, and appropriate processes put in place for the transfer, storage, restricted access, and disposal of personal information. Relevant Standard Operating Procedures, Guidelines and Work Instructions in relation to data management, processing and analysis of data will be followed. Following the end of the trial, data will be archived for a minimum of 7 years.

### Confidentiality {27}

All information collected during the course of the trial will be kept strictly confidential. Information will be held securely on paper and electronically; paper copies in locked file cabinets and electronic copies in password-protected files or databases secured with password-protected access systems. All records that contain personal identifiers, such as informed consent and contact details forms, will be stored separately from study records identified by Trial ID code.

Once the study is completed, sites will archive all study data until authorisation for confidential destruction is provided by the study sponsor. Electronic data transferred between sites, the CTRU or the University of Nottingham will be encrypted. Data will be archived at secure facilities at the University of Nottingham and University of Leeds.

### Plans for collection, laboratory evaluation and storage of biological specimens for genetic or molecular analysis in this trial/future use {33}

Not applicable, no specimens collected.

## Statistical methods

### Statistical methods for primary and secondary outcomes {20a}

A detailed statistical analysis plan will be finalised and agreed by the research team prior to analysis.

A single final analysis is planned after the trial is closed to recruitment and follow-up and when the full database has been cleaned and locked. Analyses will be completed by the CTRU statisticians using SAS version 9.4 [66].

The primary analysis will compare the proportions of participants in work/education for ≥ 80% of the their pre-injury hours 12 months post-randomisation between arms, using a two-level mixed effects logistic regression model, accounting for therapist-level clustering (in both arms) as random effects. The model will adjust for treatment allocation, data informing stratification factors (site, age, sex, time since injury and ISS; included as continuous covariates where applicable) and other relevant known predictors of the outcome as fixed effects. If a participant is unable to report their working status, e.g. if they are dead, this will be taken as they have not returned to work. Otherwise missing data will be assumed missing at random and multiple imputation will be used in primary analyses.

Analysis of secondary outcomes at 3, 6 and 12 months will use the same approach as the analysis for the primary outcome measure with the relevant model for the type of outcome variable using multi-level linear (adjusted for baseline where appropriate), logistic or Cox regression as appropriate.

Results will be expressed as point estimates (mean difference, odds ratios, hazard ratios), together with standard errors, 95% confidence intervals and *p* values. Unadjusted and adjusted ICCs will also be reported.

### Interim analysis {21b}

No formal interim analyses are planned. An internal pilot analysis focussed on recruitment figures will be performed 10 months after the first two sites have opened (for recruitment rates) and 12 months later (for retention rates), participants from this analysis will be included in the main trial*.*

### Methods for additional analyses {20b}

Descriptive data (e.g. total numbers of screened patients, patients eligible for participation, those providing consent, reasons for non-entry) will be reported and baseline characteristics for each study arm summarised. Baseline characteristics of those lost to follow-up will also be compared with those not lost to follow-up to assess for bias at each follow-up timepoint. Quantitative summaries of intervention delivery will evaluate uptake of the ROWTATE VR intervention, adherence to the processes and quality of intervention delivery. Data on usual care and adverse events will be summarised descriptively.

An exploratory Complier Average Causal Effect (CACE) analysis will be conducted on the primary endpoint in order to measure the impact of the intervention amongst participants who complied with the intervention (attendance at ≥ 70% sessions). The CACE models will use the same multi-level mixed effects modelling techniques as the primary analysis model to ensure reasonable comparisons can be made. Sensitivity analysis will be conducted to vary the definition of compliance (i.e. attendance at > 60%, > 80% sessions).

For the primary outcome, potential moderators (via an interaction between the treatment arm and potential moderator) of the treatment effect will be explored for key baseline factors (i.e. age, gender, job category, anxiety, depression, type of injury and injury severity) and mediators of the treatment effect will be explored for post-randomisation factors (i.e. number and proportion of intervention sessions attended, work/education intentions, job/education retention, sickness absence, work/education at 3/6 months, anxiety, depression, benefits claimed, financial stress and pain).

### Analysis population definitions and methods for handling missing data {20c}

Analyses will be conducted on the intention to treat (ITT) population, which includes all randomised participants, regardless of adherence to the intervention, analysed as randomised.

Missing data are expected at the item, outcome and time point levels. Where questionnaires have published scoring protocols, detailing the handling of missing item data, these will be followed. If not, missing items will be imputed as the mean of non-missing data for that item where 50% or more items for a scale are available. Mechanisms for missing outcome data will be explored and missing data will be imputed via multiple imputation. Sensitivity analysis will explore the impact of employing different missing data handling strategies, including analysis to the availability of data.

### Plans to give access to the full protocol, participant-level data and statistical code {31c}

Data will be available upon request and subject to approval by the study funder, sponsor and TSC. A data sharing agreement will be required prior to access /sharing of any study data.

#### Health economic analysis

An economic evaluation alongside the trial will be conducted to determine whether the ROWTATE VR intervention plus usual care is cost effective compared to usual care alone at 12 months post-randomisation. As recommended by NICE guidance [67] (2022), the primary health economic analysis will take a NHS and personal social services cost perspective. Secondary analysis will use a societal perspective to capture the broader effects of return to work (RTW) on out-of-pocket expenses and economic effects on the individual, carers and families notably the productivity gain from RTW and a reduction in time spent on caring for the patient. This will enable a broader societal perspective to be reported alongside a health service perspective.

A detailed micro-costing within the trial for both intervention and usual care arms will be conducted, using a patient resource proforma designed for the trial. The proforma has been designed based on a patient focus group which identified key cost drivers for trauma. It will collect patient-level resource information on all aspects of initial and follow-up resources used by the patient, NHS and community care services (Table 3) at 3, 6 and 12 months on follow-up questionnaires from all participants in both arms. A monthly calendar will be provided for participants to record daily resource use to facilitate questionnaire completion. Intervention-specific resource use will be derived from intervention CRFs (Table 3).

Resource use units from patient follow-up questionnaires and therapist CRFs will be transformed to costs applied, using national sources such as NHS and community services prices, such as the Unit Cost of Health and Social Care, Personal Social Services Research Unit (PSSRU), the British National Formulary (BNF) and the National Reference Cost Collection.

The main outcome measure will be the QALYs derived from the EQ-5D-5L questionnaire completed at baseline, 3, 6 and 12 months. These will be combined with patient and therapist reported health care and societal costs to inform the cost-utility analysis from an NHS perspective and a separate secondary analysis taking a societal perspective. No discounting will be applied to derived QALYs or costs due to their being incurred within a 12-month period. Patient responses to the EQ-5D-5L will be converted into utility scores using standard UK population tariff values. These will be transformed into QALYs using the trapezium rule to calculate the area under the curve. The value set for EQ-5D-5L has not yet been published by NICE (see 2019 official statement) [68]. Whilst there is currently controversy around the EQ-5D-5L tariff, we will map the EQ-5D-5L values back to the EQ-3D using the Crosswalk Index Value Calculator to provide consistency with current NICE requirements [69].

The economic outcomes of the cost-utility analysis are an Incremental Cost-Effectiveness Ratio (ICER), along with the incremental monetary benefit, both of which represent the monetary value to the NHS when the willingness-to-pay threshold for a specified outcome is known. Specifically, we will use the net monetary benefit framework to estimate the extent to which the intervention is a cost-effective option compared to usual care at the NICE threshold of £20,000–£30,000 per QALY gain. If the incremental net benefit is positive, then the intervention is cost effective at the desired threshold. Cost-Effectiveness Acceptability Curves (CEACs) will be constructed to show the probability that the ROWTATE VR intervention is cost effective compared to usual care across a range of willingness-to-pay thresholds. Key cost drivers will be examined using sensitivity analysis.

## Oversight and monitoring

### Composition of the coordinating centre and steering committee {5d}

The trial forms part of a programme of research which has both programme and trial level governance:

#### Programme Steering Group (PSC)

The PSC, comprising independent members with appropriate clinical and statistical expertise and an independent Chair, will provide overall supervision of the programme grant. The Chief Investigators (CIs) and other members of the Programme Management Group (PMG) will attend the PSC meetings and report progress on all the work packages. The PSC will operate in line with the Study’s Committee Terms of Reference as agreed at their first meeting.

#### Trial Steering Committee (TSC)

The Programme Steering Committee has agreed to act as the TSC. It will comprise independent members with appropriate clinical expertise, and an independent Chair will provide overall supervision of the trial, in particular trial progress, adherence to protocol, patient safety and consideration of new information. The CIs and other members of the TMG will attend the TSC meetings and present and report progress. The TSC will operate in line with the PSC Terms of Reference as agreed at their first meeting.

### Programme Management Group (PMG)

The Programme Management Group (PMG), which oversees the ROWTATE Programme Grant, comprises the CI, Co-Applicants and Co-investigators. The PMG will oversee the whole programme of studies.

#### Trial Management Group (TMG)

The TMG, comprising the CI, CTRU team, Patient and Public Involvement Group (PPI) and other key external members of staff involved in the trial and will be assigned responsibility for the clinical set-up, ongoing management, promotion of the trial and for the interpretation and publishing of the results. Specifically the TMG will be responsible for (i) protocol completion, (ii) CRF development, (iii) obtaining approval from the main Research Ethics Committee (REC) and supporting applications for Health Research Authority (HRA) Assessments, (v) completing cost estimates and project initiation, (vi) nominating members and facilitating the TSC, (vii) reporting of serious adverse events, (viii) monitoring of screening, recruitment, treatment and follow-up procedures, (ix) auditing consent procedures, data collection, trial endpoint validation and database development.

#### Patient and Public Involvement Group (PPI Group)

The PPI group comprises 15 members with lived experience of traumatic injury, or for caring for someone with a traumatic injury. The PPI group is led by a brain injury survivor and meets quarterly with the Chief Investigators and members of the research team. Two PPI members (IA, SF) sit on the TMG. PPI group members have contributed or will contribute to the trial in the following ways:

- Membership of the TMG
- Advice on patient-facing documentation
- Advice on design of recruitment strategies
- Advice on design of follow-up methods
- Advice on content of follow-up questionnaires
- Advice on adverse event data collection
- Input into selection of outcome measures
- Attendance and contribution to therapist intervention training sessions
- Reviewing follow-up rates and advice on maximising follow-up
- Interviewing patient participants and assisting with analysis of interviews in the process evaluation embedded in the trial
- Preparation of a newsletter for PPI representatives, researchers and therapists involved in the trial
- Contributing to publications and other outputs from the trial

In addition, two independent PPI representatives sit on the PSC.

### Composition of the data monitoring committee, its role and reporting structure {21a}

A subgroup of the TSC will monitor ethical and safety issues and report to the TSC. The subgroup will comprise independent members including clinicians, a statistician and a PPI representative.

### Adverse event reporting and harms {22}

As repeated hospital admissions for treatment and procedures related to the original injury are expected in this patient population, only Related and Unsuspected Serious Adverse Events (RUSAE) will be reported. These are defined as a serious adverse event that is unexpected in its severity and seriousness and deemed directly related to or suspected to be related to the trial intervention. Possible examples are accidental injury resulting from working with equipment or workplace adaptations recommended by the intervention OT or work place accidents resulting in injury requiring hospital treatment. These events will be collected by self-report via participant completed study questionnaires at 3, 6 and 12 months (Table 4) and ad hoc CRFs, or via a site notifying the CTRU. The Chief Investigator (or delegate) will be responsible for assessing if the reported event meets the definition of a RUSAE. If deemed to be a RUSAE, standard reporting procedures to the Sponsor, Research Ethics Committee and TSC will be followed and the Chief investigator will take appropriate action to minimise harm and make necessary protocol amendments.

Negative outcomes resulting from the intervention which do not meet the definition of a RUSAE (referred to as possible harms) will be collected via mentoring and recorded on the mentoring record.

### Frequency and plans for auditing trial conduct {23}

Site monitoring and audit will be informed by a risk-based approach, with quality assurance audit undertaken by, or on behalf of, the Sponsor if there is cause to do so.

### Plans for communicating important protocol amendments to relevant parties (e.g. trial participants, ethical committees) {25}

Protocol amendments requiring REC review will not be implemented until the amendment has received a favourable opinion from the REC and HRA and been accepted by the Research and Development (R&D) department of the participating site, in accordance with the guidance at the time of the amendment. In the event of an amendment to the protocol which affects participants’ involvement in the study, participants’ will be provided with updated information and continuing consent will be obtained using amended informed consent forms, which will be signed by the participant.

### Dissemination plans {31a}

On completion of the study, a final study report will be produced. This will be made available on the ROWTATE website (www.rowtate.org.uk). A summary of the study findings will be made available on the ROWTATE website. Findings will also be published in peer-reviewed journals and presented at conferences. The full study report will be published by the NIHR and made available online by the NIHR journals library.

### Protocol amendments {25}

Plans for communicating important protocol modifications (e.g., changes to eligibility criteria, outcomes, analyses) to relevant parties (e.g., investigators, REC, trial participants, trial registries, journals, regulators).

Several changes have been made to the protocol since trial registration. These are:

1. Increasing the number of trial sites from five to eight to increase recruitment to the trial
2. Extending the recruitment period from 20 to 30 months due to slower than anticipated recruitment.
3. We have made changes to the follow-up questionnaires and the administration process to improve the response rate for follow-up questionnaires collecting secondary outcome data. These decisions were made in consultation with our PPI group. We added the option for participants to complete the follow-up questionnaires by telephone and changed the use of £10 vouchers from conditional (i.e. on return of questionnaire) to unconditional (i.e. sent with questionnaire). We shortened the follow-up questionnaires by removing some measures, or by replacing longer scales with shorter versions. Discussions were held with the PPI group about which outcome measures were the least important to retain from the patients’ perspective. The follow-up questionnaires have been shortened by:
  a Removing the Work Limitations Questionnaire, the WHODAS 2.0, recovery expectations questions and questions on travel costs
  b Replacing the IES-15 with the IES-6
  c Replacing the Purpose in Life scale (9 items) with the Purpose in Life Test - Short Form (4 items)
4. We added additional secondary outcomes so that we could measure changes in hours worked (number of hours returned to work/education and the percentage of pre-injury hours returned to work/education)

## Discussion

This paper describes the protocol for a randomised controlled trial of early vocational rehabilitation and psychological support in trauma patients aimed at improving return to work. A comprehensive process evaluation and implementation study is embedded within the trial and will address therapist competence to deliver the intervention, the content and context of usual care and of the intervention, fidelity and acceptability of the intervention, implementation issues and dissemination of implementation findings. The protocol for this embedded study will be published elsewhere.

Strengths of our study include the inclusion of participants with a wide range of injuries, which should ensure findings are generalisable to trauma populations with moderately severe or major trauma. Our intervention is theoretically-based and was developed using a person-based approach (details of the intervention development process will be published elsewhere). The intervention takes a biopsychosocial approach and unlike most VR interventions for trauma patients [23–27], we have incorporated clinical psychologist support for those meeting thresholds on standard tools. The intervention should therefore impact on a range of modifiable factors which are associated with poorer occupational outcomes [70–75]. Our feasibility study has shown that the intervention can be delivered with fidelity and is acceptable both to patients and those delivering the intervention [76] (full feasibility findings will be published elsewhere). The outcomes being measured in our trial have been informed by research incorporating trauma patient perspectives [77]. We are working collaboratively with a group of patients with lived experience of traumatic injury, who have made important contributions to intervention development, trial design and trial conduct, ensuring the trial processes, intervention and findings meet the needs of injured patients.

There are several challenges to conducting this RCT. Recruitment to the trial has been slower than anticipated due to delays in site set-up, recruitment staff absences at sites where only one member of staff recruits and limited capacity of occupational therapists delivering the intervention which results in sites pausing recruitment until therapist capacity improves. The recruitment period has therefore been extended from 20 to 30 months. Funding to deliver interventions in trials in England has to be obtained separately from obtaining funding to conduct a trial. The costs of delivering the intervention, above the costs of providing usual care, are termed excess treatment costs (ETCs) [78]. These are paid by services commissioners to NHS services providing interventions. Each provider has an annual ETC threshold that must be reached before they receive any ETC payments. Obtaining agreement from NHS hospital Trusts to cover the costs of delivering the intervention has required complex and lengthy negotiations. This may make it difficult to add additional sites to the trial in a timely manner should this be required.

We have previously shown that rehabilitation pathways in English MTCs are complex and vary considerably between centres and that there are gaps in service provision [33]. This poses practical issues in terms of availability of therapists working within the same NHS Trust as the MTC to deliver the intervention. This has been exacerbated by the Covid-19 pandemic, with staff shortages, sickness absence, re-deployment to Covid-related work and in the case of clinical psychologists, requirements to provide additional support to NHS staff [79]. This impacts on site set-up as new and different models for contracting therapists to provide the intervention at different NHS Trusts need to be developed, and complex information governance and research governance issues arise. The time taken to resolve these issues makes it difficult to add additional sites to the trial in a timely manner should this be required. In addition, the time taken to address these issues makes it difficult to address therapist capacity issues at recruiting sites in a timely manner, so limiting recruitment at sites where therapist capacity is low.

The ROWTATE VR intervention was initially developed as a face-to-face intervention [80] and was adapted to be delivered remotely at the start of the Covid-19 pandemic. The feasibility of delivering the remote intervention was tested in a small non-randomised single-arm feasibility study, whose findings will be presented elsewhere. The feasibility study was conducted during the Covid-19 pandemic, which restricted recruitment strategies, limited the number of participants and the scope of the study. The feasibility study was therefore unable to adequately test recruitment and follow-up strategies. The internal pilot within the RCT will provide some data on the effectiveness of these strategies, but ideally these would have been tested in a feasibility study prior to the trial.

Our trial is individually randomised and there is therefore potential for contamination, whereby control arm participants receive the intervention, or some elements of the intervention. [81] Contamination dilutes the treatment effect, with the difference between the intervention and usual care groups tending towards null. Our trial has multiple strategies in place to minimise contamination including specific and repeated training on contamination for therapists, restricting access to training materials and intervention resources, notifying therapists delivering the intervention of usual care participants so that they do not treat these patients and identifying possible contamination risks and strategies to minimise these in mentoring sessions. Despite this, it is possible that some contamination may occur, and any instances will be recorded and reported.

We are measuring outcomes at 3, 6 and 12 months. Whilst most trauma patients who are going to return to work will do so within 12 months of their injury, there is some evidence that a small percentage of patients will return to work later than this [72, 82]. Longer-term follow-up of our trial participants would therefore be useful and sources of support for this will be investigated.

This trial will help address the lack of evidence regarding VR interventions for moderately severe and major trauma populations. Evidence of a clinically and cost-effective VR intervention will be important for commissioners and providers to enable adoption of VR services for this large and important group of patients within the NHS.

## Trial status

Protocol version 7.0, 24/10/23. Recruitment for the study commenced in October 2021 and is planned for 30 months.

### Supplementary Information

Supplementary Material 1. Participant information sheet and informed consent form.

Supplementary Material 2. Tables of baseline and follow-up assessments and box of internal pilot progression criteria.

Supplementary Material 3. REC approval letter.

Supplementary Material 4. Original funding documentation.

## Data Availability

To maintain the scientific integrity of the study, data will not be released prior to the end of the study, either for study publication or oral presentation purposes, without the permission of the TSC. Access to data or sharing data with third parties will be subject to approval by the study funder, sponsor and TSC. A data sharing agreement will be required prior to access/sharing of any study data.  Data will be kept for a minimum of 7 years following the end of the trial. During the archiving period (i.e. after the TSC has been disbanded at the end of the trial and before destruction of data), any requests for access to or copies of data will be considered by all collaborators in consultation. The sponsor will be the final arbiter of whether any disclosure/sharing should be agreed.

## Abbreviations

AE: Adverse event
BNF: British National Formulary
CACE: Complier Average Causal Effect
CEA: Cost-effectiveness analysis
CI: Confidence interval
CEACs: Cost-Effectiveness Acceptability Curves
CP: Clinical psychologist
CRF: Case report form
CTRU: Clinical Trials Research Unit
CUA: Cost-utility analysis
EoI: Expression of Interest
EQ5D-5L: EuroQol 5_Dimension Health Questionnaire 5-level
GAD-7: 7-Item Generalised Anxiety Disorder Assessment
GP: General Practitioner
HRA: Health Research Authority
ICC: Intraclass correlation coefficient
IES-15: Impact of Event Scale (15-item scale)
IES-6: Impact of Event Scale (6-item scale)
ISS: Injury Severity Score
ISRCTN: International Standard Randomised Controlled Trials Number
ITT: Intention to treat analysis
MoCA: Montreal Cognitive Assessment
MTC: Major Trauma Centre
NHS: National Health Service
NICE: The National Institute for Health and Care Excellence
NIHR: National Institute of Health Research
OT: Occupational therapist
PSSRU: Personal Social Services Research Unit
PHQ-9: 9-Item Patient Health Questionnaire
PI: Principal Investigator
PIS: Participant Information Sheet
PMG: Programme Management Group
PSC: Programme Steering Committee
PTSD: Post-traumatic stress disorder
QALY: Quality-adjusted life year
QoL: Quality of Life
R&D: Research and Development
RCT: Randomised controlled trial
REC: Research Ethics Committee
RTW: Return to Work
RUSAE: Related Unexpected Serious Adverse Event
SAE: Serious adverse event
SD: Standard deviation
TMG: Trial Management Group
TOSCE: Team Objective Structured Clinical Examination
TSC: Trial Steering Committee
UC: Usual care
WAI: Work Ability Index
WHODAS: World Health Organisation Disability Assessment Schedule
UK: United Kingdom
