# Supplementary material for: Early vocational rehabilitation and psychological support for trauma patients to improve return to work (the ROWTATE trial): study protocol for an individually randomised controlled multicentre pragmatic trial
Source: Trials. 2024 Jul 2;25:439. doi: 10.1186/s13063-024-08183-w (PMC11221047; doi:10.1186/s13063-024-08183-w)

**Delete this line, then print on Trust/Hospital headed paper**

**
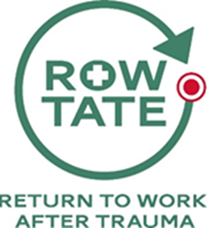
`**

| Participant ID: | Initials: |
| --- | --- |
| Date of Birth: | NHS/Hospital Number: |
| Principal Investigator: | |

**ROWTATE** – **Return To Work After Trauma**

**PART 1: PARTICIPANT CONSENT FORM**

|  | **The following statements are MANDATORY**  You need to agree to all of them to take part in the study | ***Please initial each box*** |
| --- | --- | --- |
| 1 | I confirm that I have read and understand the Participant Information Sheet dated XX/XX/XXXX (Version X.0) for the above study. I have had the opportunity to consider the information, ask any questions and have had these answered satisfactorily. |  |
| 2 | I understand that my participation in this study is voluntary and that I am free to withdraw at any time without giving any reason, without my medical care or legal rights being affected. |  |
| 3 | I understand that even if I withdraw from the study, the data collected from me will be used in analysing the results of the study |  |
| 4 | I understand that relevant sections of my healthcare/medical records and data collected during the study may be looked at by authorised individuals from the research team, the Nottingham University Hospitals NHS Trust (the study sponsor), the NHS Trust and regulatory authorities where it is relevant to my taking part in this research. I give permission for these individuals to have access to my records. |  |
| 5 | I understand that the information collected about me may be used to support other research in the future. This may be shared with other researchers and wherever possible any information that could identify me will be removed |  |
| 6 | As part of this research I understand that a study researcher may wish to observe some of the support I receive and I may be asked if I would like to discuss my experiences of the treatment I have received with a researcher. |  |
| 7 | I understand that my postal address and/or e-mail address and telephone numbers will be passed to the Research Office (at the University of Leeds) for the purpose of completing the questionnaire booklets. |  |
| 8 | I agree for my details and a copy of this consent form (which will include my name and date of birth) to be stored by the Research Office (at the University of Leeds) for the purposes of this study. |  |
| 9 | I agree to my General Practitioner (GP), or any other doctor treating me, being informed of my participation in this study. I agree to a copy of this Consent Form being sent to my GP. |  |
| 10 | I agree to take part in the above study. |  |

**Participant**

|  | |
| --- | --- |
|  | |
| Day / Month / Year    ……….…../……….…/…….…… |  |

Signature:

Name (block capitals):

Date:

**Witness (if required)**

|  | |
| --- | --- |
|  | |
| Day / Month / Year    ……….…../……….…/…….…… |  |

Signature:

Name (block capitals):

Date:

**Person taking consent**

I have explained the study to the above named participant and he/she has indicated his/her willingness to participate.

|  | |
| --- | --- |
|  | |
| Day / Month / Year    ……….…../……….…/…….…… |  |

Signature:

Name (block capitals):

Date:

(1 copy for patient; 1 for the CTRU; 1 held in patient notes, original stored in Investigator Site File)


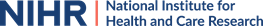

Supplement: Supplementary file 1 — Supplementary Material 1. Participant information sheet and informed consent form. [file 13063_2024_8183_MOESM1_ESM.docx]
