# Supplementary material for: Early vocational rehabilitation and psychological support for trauma patients to improve return to work (the ROWTATE trial): study protocol for an individually randomised controlled multicentre pragmatic trial
Source: Trials. 2024 Jul 2;25:439. doi: 10.1186/s13063-024-08183-w (PMC11221047; doi:10.1186/s13063-024-08183-w)
Supplement: Supplementary file 2 — Supplementary Material 2. Tables of baseline and follow-up assessments and box of internal pilot progression criteria. [file 13063_2024_8183_MOESM2_ESM.docx]

Table 1. Baseline assessments

| **Assessment** | **Type** | **Method of Completion** |
| --- | --- | --- |
|  |  |  |
| Participant demographics | Questionnaire booklet | Researcher/self-completion |
| Contact details | CRF | Researcher |
| Injury details | CRF | Researcher |
| Montreal Cognitive Assessment (MoCA) | CRF | Researcher |
| Work/education status | Questionnaire booklet | Researcher/self-completion |
| Health related quality of life (EuroQol EQ-5D-5L) | Questionnaire booklet | Researcher/self-completion |
| Patient Health Questionnaire (PHQ-9) | Questionnaire booklet | Researcher/self-completion |
| Generalised Anxiety Disorder Assessment (GAD-7) | Questionnaire booklet | Researcher/self-completion |
| Impact of events scale (15 items) | Questionnaire booklet | Researcher/self-completion |
| World Health Organisation Disability Assessment Schedule 2.0 (12 items) | Questionnaire booklet | Researcher/self-completion |
| Recovery expectations | Questionnaire booklet | Researcher/self-completion |
| Financial Chronic Stress Scale (3 items) | Questionnaire booklet | Researcher/self-completion |

CRF=Case report form

Table 2. Follow-up assessments

| **Assessment** | **Type** | **Method of Completion** | **3 Months** | **6 Months** | **12 Months** |
| --- | --- | --- | --- | --- | --- |
| Discharge Information (including final ISS score) | CRF | Researcher | Quarterly | | |
| Work/Education status | Questionnaire booklet | Researcher / Self-completion | X | X | X |
| Health Related Quality of Life (EuroQol EQ-5D-5L) | Questionnaire booklet | Researcher / Self-completion | X | X | X |
| Patient Health Questionnaire (PHQ-9) | Questionnaire booklet | Researcher / Self-completion | X | X | X |
| Generalised Anxiety Disorder Assessment (GAD-7) | Questionnaire booklet | Researcher / Self-completion | X | X | X |
| Impact of Events Scale (6 item scale) | Questionnaire booklet | Researcher / Self-completion | X | X | X |
| Work Ability Index (WAI item 1 (workability scale) WAI item 2  (physical and mental demands) | Questionnaire booklet | Researcher / Self-completion |  |  | X |
| Financial Chronic Stress Scale (3 item scale) | Questionnaire booklet | Researcher / Self-completion | X | X | X |
| Purpose in life test - short form scale (4 items) | Questionnaire booklet | Researcher / Self-completion |  |  | X |
| Health and Social Care Resource Use (inpatient and outpatient hospital visits, primary and community care use, medication, aids and adaptations, informal care) | Questionnaire booklet | Researcher / Self-completion | X | X | X |
| Intervention Resource Use (intervention session content and additional activity forms recording therapist’s direct time and indirect time (time travelling to/from treatment appointments and administrative time) | CRF | Occupational therapists and clinical psychologists | Throughout intervention period | | |
| Safety Reporting (workplace accidents requiring medical attention, workplace accidents involving equipment or adaptations to work environment, injuries received, healthcare used) | Questionnaire booklet | Researcher / Self-completion | X  (only if RTW) | X (only if RTW) | X (only if RTW) |

RTW=Return to work

Box 1. Progression criteria for the internal pilot

- Green (go): mean of more than 6 participants recruited per month per site;
  At least 80% of participants complete the primary outcome
- Amber (modify): mean of 4-6 participants recruited per month per site;
  60%-79% of participants complete the primary outcome
- Red (stop): mean of less than 4 participants per month per site;
  Less than 60% of participants complete the primary outcome.
