# Supplementary material for: Early vocational rehabilitation and psychological support for trauma patients to improve return to work (the ROWTATE trial): study protocol for an individually randomised controlled multicentre pragmatic trial
Source: Trials. 2024 Jul 2;25:439. doi: 10.1186/s13063-024-08183-w (PMC11221047; doi:10.1186/s13063-024-08183-w)
Supplement: Supplementary file 4 — Supplementary Material 4. Original funding documentation. [file 13063_2024_8183_MOESM4_ESM.pdf]

National Institute for Health Research

Central Comissioning Facility
Grange House
15 Church Street
Twickenham
TW1 3NL

Tel:020 8843 8000
Fax:020 8843 8001
Email:ccf@nihr.ac.uk
Web: www.nihr.ac.uk/ccf

Professor Denise Kendrick
University of Nottingham
Primary Care, Floor 13
Tower Building University Park
Nottingham
Nottinghamshire
NG7 2RD

23 April 2018

Dear Professor Kendrick and Dr Kathryn Radford,

NIHR Programme reference number RP-PG-0617-20001: Multicentre Research Programme to Enhance Return to Work after Trauma (ROWTATE)

I am pleased to inform you that the panel has recommended your application submitted for consideration in Competition 24 for funding and the Department of Health and Social Care, in their capacity as the National Institute for Health Research (NIHR), has confirmed their intention to award funding upon acceptance of the terms and conditions set out in the Standard Research Contract and pending agreement to the suggested amendments recommended by the panel, as detailed in the accompanying document.

The Standard Research Contract, between Contractors and the Secretary of State for Health and Social Care for all initiatives can be found on the NIHR website at:

https://www.nihr.ac.uk/funding-and-support/funding-for-research-studies/manage-my-study/contracts-and-intellectual-property.htm.

Next Steps

The NIHR is committed to the rapid initiation of research following the decision to fund to benefit patients as soon as possible. Therefore, we expect funded researchers to be working towards gaining the necessary contractual agreements and governance approvals required to start the project by a date mutually agreed by both parties on acceptance of the award.

The NIHR acknowledges the risk to organisations around committing resource to research before a contract is in place; however, it is rare to not reach contractual terms unless the circumstance of the research team changes. The NIHR, therefore, encourages organisations to commit staff to setting up projects at as early an opportunity as possible in order to expedite the formal commencement of research.

It is acknowledged that there can be unforeseen delays in starting up a research project, but in order to help reduce these it is your responsibility to work closely with your organisation's R&D department or equivalent as well as other colleagues / departments involved in the administration and management of the research, and to start these discussions at the earliest opportunity.

To ensure that the project starts within the agreed timeframe with all the required agreements and approvals in place, appropriate staff (such as project and/or study managers) need to be in post as early as possible after receiving this letter of intent. These staff costs will ultimately be covered through the research funding award, but you are encouraged to meet them from Research Capability Funding (RCF) prior to the research contract being agreed.

To support the often-iterative process towards agreement of the contract, we have set out the guiding timeframes for the submission of responses or information for each step towards the agreement of the Standard Research Contract as well as the anticipated start date.

- Confirmation of acceptance of funding – no later than 1 week from date of letter
- Responses to Panel feedback and queries – no later than 2 weeks from date of letter
- Responses to Finance and IP queries – 2 weeks from date of letter
- Submission of draft collaboration agreements and/or subcontracts (where applicable) – Prior to the start date or at a mutually agreed date
- Contract signature – 6 months from date of letter
- Contracted commencement start – 3 months from contract signature or a mutually agreed date

On receipt of information as set out above, the NIHR through the Central Commissioning Facility is committed to responding to your submission of information within two weeks or we will update you on progress.

Please take the time to carefully read the enclosures to this letter which details the feedback on your application, the processes to be undertaken during the next steps, as well as additional information relating to your award.

Yours sincerely,

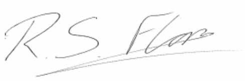

Rajinder Flora
Assistant Director, Programme Grants for Applied Research

cc: Dr Maria Koufali, Deputy Director Research & Innovation

Additional Information

Publicity
We must remind you to refrain from publicising this funding recommendation until negotiations have been completed and a contract has been signed.

Response to feedback
Your response to the concerns of the sub-panel should form no more than 6 pages. An annex (e.g. for references, diagrams, tables *etc.*) of no more than 10 pages is permitted. Where applicable, your response to finance and intellectual property queries should each form no more than 6 pages. Annexes are not permitted.

Once you have completed all the relevant steps above, please email your completed response to programme.grants@nihr.ac.uk.

Intellectual Property
In order to expedite the IPR and Warranties and Liabilities terms and conditions, please clarify with local R&D management whether there are any third party rights in the background IP which may affect the research, as these will need to be stated clearly in schedule C of the contract. Please also ensure that your plans for managing the foreground IP are in line with the local IPR policy of the host organisation. As described in condition 15.2, our preferred position is that the foreground IP of this research project shall vest in the contractor, given that an NHS body or other provider of NHS services is best placed to realise any patient benefits flowing from the research. We will request clarification on all of these issues during the contractual negotiations. Please note that the terms and conditions of this contract are subject to review and amendment at the discretion of DH. **An overview of contractual responsibilities can be found in *Annex 2*.**

Notification of start date
In order to ensure that payments for research projects are not made in advance of need, PGfAR will require confirmation of the actual start date.

The PM will email the R&D contact (copying in the Chief Investigator) for the award to officially confirm the start date on behalf of the host organisation.

DH wishes to see a dramatic and sustained improvement in the initiation of clinical research. In the event that the actual start date is more than three months after the contracted start date, the host organisation will be required to sign a variation to contract that will be issued by the PM. Please be aware that a drop dead date (six months after the estimated start date) is included within the contract to prevent serious delays.

Clinical Research Network (CRN)
If your study involves the NHS or NHS patients we expect you to apply, where appropriate, for consideration for CRN support in England and/or its equivalents in the devolved administrations and subsequent inclusion in the CRN Portfolio Database. If your study is deemed eligible for consideration for CRN support in England and/or its equivalents in the devolved administrations we expect you to:

- Keep your study record on the Portfolio Database up to date.
- Upload your recruitment data into the Portfolio Database on a monthly basis. Please note that the CRN will share this data with us through the production of quarterly reports.

Information on the CRN support available to researchers can be found at http://www.nihr.ac.uk/funding-and-support/documents/Study-Support-Service/CRN_SSS_leaflet_Sept16_forweb.pdf and information about gaining NHS permission for clinical research and using the NIHR Coordinated System for gaining NHS Permission (CSP) can be found at http://www.nihr.ac.uk/funding-and-support/documents/Study-Support-Service/CRN_support_routemap_2016_final.pdf.

Clinical trials
All primary research studies should also be assigned an International Standard Randomised Controlled Trial Number (ISRCTN). You can find further information at the ISRCTN website at www.isrctn.org. Please note that

the remit of this database has been widened to include all primary research projects, even those that are not randomised controlled trials. There is no registration fee for NIHR funded trials and it is advised that you register your trial prior to initiation of patient recruitment.

Finally, we must remind you again to refrain from publicising this funding recommendation until negotiations have been completed and a contract has been signed.

IRAS

HRA Approval is for all project-based research that involves NHS organisations in England where the NHS organisation has a duty of care to participants, either as patients/service users or NHS staff/volunteers. References to participants include people whose data or tissue is involved in a research project.

It brings together the assessment of governance and legal compliance, undertaken by dedicated HRA staff, with the independent ethical opinion by a Research Ethics Committee (REC) so that you only need to submit one application.

This page provides an overview of the HRA Approval process. For detailed guidance, go to the Integrated Research Application System (IRAS).

A series of top tips have been created to offer support with writing and submitting applications for HRA approval; these can be found in the IRAS help section. We also recommend you visit the research planning section for help before you begin your IRAS application.

Annex 2

Overview of Provider's contractual responsibilities

Below is an overview of some of your responsibilities as the holder of a Programme Grant award. This is not an exhaustive list; for a full list of the terms and conditions please refer to the standard agreement (https://www.nihr.ac.uk/funding-and-support/funding-for-research-studies/manage-my-study/contracts-and-intellectual-property.htm), and subsequently the agreed contract for your award.

1. Completion of progress reports

Award holders must submit annual progress reports throughout the duration of the programme. Template progress report forms will be made available either by email or through CCF's online research management system (RMS) https://ccfrms.nihr.ac.uk/Login. The report is designed to capture any material changes to the research or staffing, a summary of progress to date, progress against the project milestones, a list of presentations and publications, background and foreground intellectual property (IP) positions and matters requiring further attention by the PM.

Please note, NIHR reserves the right to ask for *ad hoc* reports or information in specific circumstances.

2. Variations to agreement

If at any time it appears likely that the contract, in particular the research, needs to be varied, please notify the PM as soon as possible about the nature and rationale for the anticipated change. The PM may ask you to complete an extension request form which may be sent for review by one or more of the Programme's authorities.

If the variation is granted, a formal variation to contract will be issued for signing by an authorised signatory (typically Chief Executive or Chief Financial Officer) to capture the changes.

3. Provide 28 days advance notice of publications

To keep the Department of Health in step with the dissemination of NIHR research, all chief investigators (whether an individual researcher or a research team) should observe the '28-day rule'. This currently involves submitting an electronic copy of the proposed research output, as it will be issued, to the PM responsible for your award, at least 28 days before it is published. NIHR should also receive full citations of research outputs when these become available.

If you are unsure as to whether you should inform us about a particular type of output, please seek your PMs guidance. Please note that the 28-day rule also applies to news releases to be issued by your host institution, e.g. university, NHS Trust or hospital. All research reports issued by individual researchers and/or research teams should:

- credit the NIHR as a funding organisation
- carry the NIHR disclaimer (see below).

NIHR disclaimer example:

This report/article presents independent research funded by the National Institute for Health Research (NIHR) under the Programme Grants for Applied Research programme* ***[insert programme reference number]****. The views expressed in this publication* are those of the author(s) and not necessarily those of the NHS, the NIHR or the Department of Health.*

*Insert type of output as appropriate.

Award holders are also reminded of NIHR's support for the principle of Open Access to research as set out in its statement supporting Euro PubMed Central:

http://www.nihr.ac.uk/funding-and-support/funding-for-research-studies/how-to-apply/support-for-study-teams/publishing-your-research/nihr-open-access-policy.htm

4. Completion of a final report

In order to promote the data and results of its funded research, the NIHR has created the Journals Library. The Journals Library will help disseminate the findings of the research commissioned by a subset of its funding schemes and will provide an important permanent and comprehensive record of the work which has been funded.

All Programme Grant research should be published in the Journals Library in the form of a final report. The report must be submitted **14 days** after the contract end date.

Further information regarding the format of the final report can be found on the Journals Library website (http://www.journalslibrary.nihr.ac.uk/), from which the *Information for Authors* guidance document can also be downloaded (http://www.journalslibrary.nihr.ac.uk/authors).

Under the terms of the contract, the Authority has been granted rights, on a non-exclusive basis, to publish your final report and the information contained therein. It is therefore essential that when seeking to publish your research in an appropriate peer reviewed journal, you only enter into **non-exclusive** copyright arrangements.

Most journals have suitable non-exclusive licences for government-funded research but if you do, in error, sign an exclusive copyright agreement with a publisher, it is your responsibility to alert both the publisher and your PM as soon as possible.

5. Provision of financial information (spend to date) to permit reconciliation on request

Award holders will be required to submit an annual financial statement detailing programme spend to date. Where total expenditure varies from the amount of funding awarded in any given financial year, justification for this deviation, along with plans for the recovery of any over/under spend must be provided. Where there is a significant over/under spend, further action may be taken, including revision of payment schedules or return of unspent income to the NIHR. A final financial statement will also be due on completion of the programme.

Please note that the host organisation is required to maintain proper financial records relating to the research at all times during the research period and for six years after the project's end. The Department of Health reserves the right to request further financial information about projects funded through the NIHR programmes at any time.

6. Exploitation of IP

Award holders are expected to be aware of the terms and conditions in relation to the IP rights of the research contract. You should work with the host organisation to identify, protect and maintain IP in accordance with their standard institutional IP policy. In particular, you are required to inform the PM of any results which are capable of being exploited either by direct adoption into the healthcare service or via commercialisation. The host organisation will also need to seek prior written approval from CCF if it wishes to use a third party (excluding professional advisors) to carry out exploitation activities with respect to the foreground IP funded through this award. CCF is interested in following up on the impact of research funding, so further information on directly exploitable IP arising from your project may be requested once the project is complete.
