# Supplementary material for: Early vocational rehabilitation and psychological support for trauma patients to improve return to work (the ROWTATE trial): study protocol for an individually randomised controlled multicentre pragmatic trial
Source: Trials. 2024 Jul 2;25:439. doi: 10.1186/s13063-024-08183-w (PMC11221047; doi:10.1186/s13063-024-08183-w)
Supplement: Supplementary file 3 — Supplementary Material 3. REC approval letter. [file 13063_2024_8183_MOESM3_ESM.docx]

Dear Editor,

**Re: TRLS-D-23-00537. Early vocational rehabilitation and psychological support for trauma patients to improve return to work (the ROWTATE trial): study protocol for an individually randomised controlled multicentre pragmatic trial**

Thank you for asking us to revise and resubmit this manuscript. As you are aware reviewer 2’s comments relate to another author’s paper and not to our paper. Following communication with your team, we were asked to revise and resubmit our paper and respond only to reviewer 1’s comments.

Reviewer 1 made only one comment:

*My one comment would be on recruitment. Recruitment began in Oct 21 and planned for 20 months. Please confirm if recruitment has completed or still ongoing. If completed, this should be stated in the manuscript, if ongoing a sentence or 2 on any recruitment delays and likely recruitment end date (as according to initial timelines recruitment should be completed by now?).*

We have addressed this comment in the following ways:

1. Under the recruitment section on page 23 we have added the following text:

*Due to slower than anticipated recruitment, the recruitment target was subsequently amended to 32 participants per month across all eight MTCs and the recruitment period extended to 30 months, with an anticipated recruitment end date of 31/3/2024.*

2. Under protocol amendments section on page 38 we have added:

*Extending the recruitment period from 20 to 30 months due to slower than anticipated recruitment.*

3. Under the discussion section on page 40 we have added the following text:

*Recruitment to the trial has been slower than anticipated due to delays in site set-up, recruitment staff absences at sites where only one member of staff recruits and limited capacity of occupational therapists delivering the intervention which results in sites pausing recruitment until therapist capacity improves. The recruitment period has therefore been extended from 20 to 30 months.*

4. Under the trial status section on page 42 we have amended the text to read:

*Protocol version 7.0, 24/10/23. Recruitment for the study commenced in October 2021 and is planned for 30 months.*

We hope that our revised manuscript is now suitable for publication in *Trials*.

Yours sincerely,

Professor Denise Kendrick, on behalf of all authors.
